# A dataset comprised of binding interactions for 104,972 antibodies against a SARS-CoV-2 peptide

**DOI:** 10.1038/s41597-022-01779-4

**Published:** 2022-10-26

**Authors:** Emily Engelhart, Ryan Emerson, Leslie Shing, Chelsea Lennartz, Daniel Guion, Mary Kelley, Charles Lin, Randolph Lopez, David Younger, Matthew E. Walsh

**Affiliations:** 1 A-Alpha Bio, Inc., Seattle, WA USA; 2 Massachusetts Institute of Technology Lincoln Laboratory, Lexington, MA USA; 3 Present Address: Department of Environmental Health and Engineering, Johns Hopkins Bloomberg School of Public Health, Baltimore, MD USA

**Keywords:** Machine learning, Antibody therapy, Immunological techniques

## Abstract

The dataset presented here contains quantitative binding scores of scFv-format antibodies against a SARS-CoV-2 target peptide collected via an AlphaSeq assay that can be used in the development and benchmarking of machine learning models. Starting from three seed sequences identified from a phage display campaign using a human naïve library, four sets of 29,900 antibodies were designed *in silico* by creating all *k* = 1 mutations and random *k* = 2 and *k* = 3 mutations throughout the complementary-determining regions (CDRs). Of the 119,600 designs, 104,972 were successfully built in to the AlphaSeq library and target binding was subsequently measured with 71,384 designs resulting in a predicted affinity value for at least one of the triplicate measurements. Data include antibodies with predicted affinity measurements ranging from 37 pM to 22 mM. To our knowledge, this dataset is the largest, publicly available dataset that contains antibody sequences, antigen sequence and quantitative measurements of binding scores and provides an opportunity to serve as a benchmark to evaluate antibody-specific representation models for machine learning.

## Abstract (summary)

**Table:** 

Measurement(s)	Antibody Binding
Technology Type(s)	AlphaSeq
Factor Type(s)	Antibody sequence
Sample Characteristic - Organism	Homo sapiens

## Background & Summary

Protein modelling is an area of machine learning research that has attractive potential benefits to protein engineering. Given the magnificent size of the design space for a given protein, it is not feasible to measure phenotypic properties of all possible designs empirically. Machine learning methods can help constrain the design space and serve as a basis for recommending designs to test in the lab to save time and reduce cost^1^. Such approaches have been taken to engineer enzymes^2^, fluorescent proteins^3^, and antibodies^4^ among others^5,6^. Successful examples of machine learning-enabled protein engineering have relied on access to large, labelled datasets of protein sequences that are typically generated as part of high throughput experimental campaigns. When datasets and models have been made publicly available, the entire field has benefited from ensuing comparisons and benchmarks^1,7^.

Although these approaches have been demonstrated for the engineering of antibodies, there remains a scarcity of labelled data available in the public domain to advance this area of research with respect to antibody binding. Large-scale curation efforts have resulted in databases of well over a billion antibody sequences without target or binding affinity values^8^. Other efforts have resulted in datasets with close to one thousand antibodies with labels – either target sequences^9^ or neutralization values^10^. Additional work in antibody binding prediction reports a subset of the data generated and used^4^. Outside of antibody binding, one group has published multiple manufacturability measurements for over 100 antibodies that have completed or advanced through the FDA approval pipeline^11–13^.

This lack of labelled data may be the result of differences in data requirements for training machine learning models compared to finding a design with the target phenotype in the absence of machine learning. Phage display, for example, is intended to provide the researcher with information on a small number of the top binders from a pool of >10^6^ designs^14,15^. This type of data is unsuitable for training models because poor binding sequences are unknown and no quantitative binding measurements are generated. Methods that do provide quantitative measurements, such as enzyme-linked immunosorbent assays^16^ and surface plasmon resonance^17^, rely on isolation of individual antibodies and therefore are significantly lower throughput and more expensive.

Recent methods using engineered yeast expression systems and next generation DNA sequencing overcome some of these challenges and can generate large scale datasets of quantitative protein-protein binding interactions including antibody-antigen interactions^18,19^. We utilized such a technique, referred to as AlphaSeq, to generate the dataset described here. Importantly, we first conducted a phage display experiment to identify three candidates that bind to our target, a conserved peptide in coronaviruses. Those candidates were the seed sequences and in addition to designing all single mutants, we performed *in silico* randomization to introduce two or three random mutations in the complementary determining regions (CDRs) for 119,600 designs. As a result, we generated a dataset that covers a considerable breadth of sequence space and has a wide range of binding measurements that is suitable for training machine learning algorithms.

## Methods

**Fig. 1:**
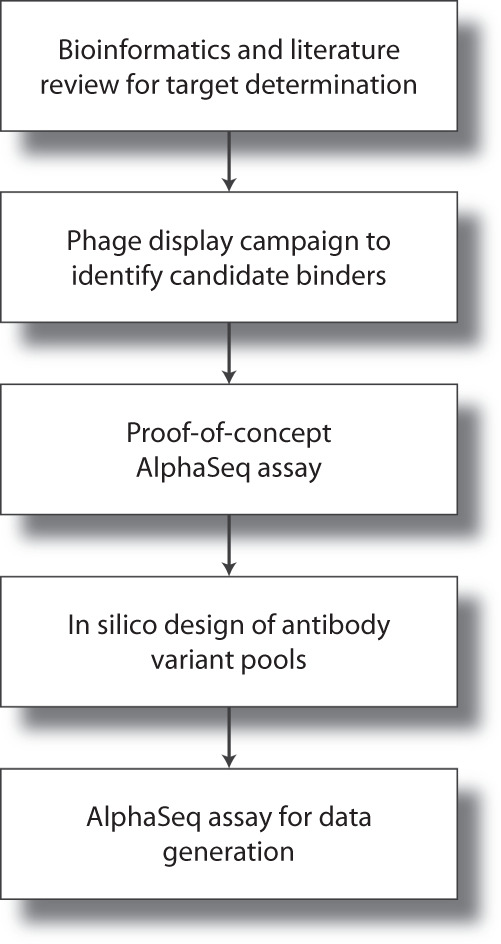
Experimental workflow for the generation of the AlphaSeq data set. A SARS-CoV-2 target peptide was identified and used in a phage display campaign to identify candidate antibodies. These antibodies were then validated for compatibility with the AlphaSeq assay. Variant antibody pools were designed using validated candidates as seed sequences and then measured in the AlphaSeq assay.

Figure 1 provides an overview of the experimental workflow.

### Target selection

Antibodies were targeted against a peptide in the HR2 region of the SARS-CoV-2 spike protein to which neutralizing antibodies have been observed^20^. Additionally, this sequence is reported to have low variability across coronaviruses and could maintain therapeutic value against viral variants^21^. The exact amino acid sequence targeted was PDVDLGDISGINAS.

### Phage display

A phage display panning experiment was performed by GenScript USA Inc. to identify candidate binders. A biotinylated target peptide, LCBiot-PDVDLGDISGINAS-OH, (vivitide, LLC) was provided to GenScript USA Inc. The human naïve phage library used by Genscript USA Inc. is marketed to be derived from 300 healthy human donors, has a size of 1.1 × 10^10^ and is in Fab format.

### AlphaSeq antibody screening

A total of five antibody sequences in scFv format were evaluated in a proof-of-concept AlphaSeq experiment; three of those five sequences bound to the target and were carried forward. All five antibody sequences were tested in both heavy-light (HL) and light-heavy (LH) chain orientation. In general, chain orientation had little to no effect on binding affinity with all but Ab-91-HL (3.39 nM) resulting in predicted K_D_ values below 1 nM. The best chain orientation was selected for each antibody; HL was selected for Ab-14, LH was selected for Ab-91 and HL was selected for Ab-95.

### In silico design of antibody libraries

Two heavy chains and two light chains were arbitrarily selected from the three antibody seed sequences listed in Table 1 for the design of the antibody library: Ab-14-VH, Ab-91-VH, and Ab-14-VL, Ab-95-VL, respectively. The goal of the in silico design process was to generate 29,900 sequence variants for each chain out of the 120,000 total sequence budget, leaving 400 sequences for controls allocated for the binding experiment. *K*-point mutations, where *k* = *1, 2*, and 3, were produced for the CDRs of each chain. Point mutations were limited to amino acid substitutions; indels were avoided to ensure the amino acid sequence was constant length. Up to *k* = 3 mutations were chosen to guarantee there was at least one instance where one amino acid substitution occurred in all CDRs of a given chain at a given time. Martin Lab’s CDR rule set was applied to extract the CDRs from their approximate positions in each chain. The number of variants for *k* = 1 mutations was determined based on the combined number of amino acid positions in the CDRs of a given chain and the total number of possible amino acid substitutions in each position. All *k* = 1 variants were kept, ensuring that duplicates and original chain sequences were removed. Using the number of sequence variants for *k* = 1, a scaling factor of ~6 was applied to determine the number of variants to sample from the total number of *k* = 2 and *k* = 3 possible sequence variants, as shown in Table 2.

**Table 1:** Target and Antibody Seed Sequences. CDRs in **bold**.

Target	PDVDLGDISGINAS
Seed ID	Antibody Sequences
14-VH	EVQLVETGGGLVQPGGSLRLSCAAS**GFTLNSYGIS**WVRQAPGKGPEWVSV**IYSDGRRTFYGDSV**KGRFTISRDTSTNTVYLQMNSLRVEDTAVYYCAK**GRAAGTFDS**WGQGTLVTVSS
14-VL	DVVMTQSPESLAVSLGERATISC**KSSQSVLYESRNKNSVA**WYQQKAGQPPKLLIY**WASTRES**GVPDRFSGSGSGTDFTLTISSLQAEDAAVYYC**QQYHRLPLS**FGGGTKVEIK
91-VH	EVQLVESGGGLVQPGRSLRLSCAAS**GFTFDDYAMH**WVRQAPGKGLEWVS**GISWNSGSIGYADSVK**GRFTISRDNAENSLYLQMNSLRAEDTALYYCAK**VGRGGGYFDY**WGQGTLVTVSS
91-VL	QAVLTQPSSLSASPGASVSLTC**TLRSGINVGTYRIY**WYQQKPGSPPQ**YLLRYKSDSDKQQGSGV**PSRFSGSKDASANAGILLISGLQSEDEADYYC**MIWHSSAWV**FGGGTKLTVL
95-VH	EVQLVESGAEVKKPGASVKVSCKAS**GYTFTSYGIS**WVRQAPGQGLEWMGW**ISAYNGNTNYAQ**KLQGRVTMTTDTSTSTAYMELRSLRSDDTAVYYCAR**VGRGVIDH**WGQGTLVTVSS
95-VL	SSELTQDPAVSVALGQTVRITC**EGDSLRYYYAN**WYQQKPGQAPILVIY**GKNNRPS**GIADRFSGSNSGDTSSLIITGAQAEDEADYYC**SSRDSSGFQVF**FGAGTKLTVL

**Table 2:** Distribution and incorporation of mutations by library and *k* mutations. Additionally, there are seven seed sequences with no mutations.

Library	ScFv Seed	Scaling Factor	*k* mutations	No. Sequences Designed	No. Sequences Present	% Present Per k mutations	% Present (Overall)
AAYL49	14 Heavy	6.06	1	665	594	89%	88.5%
			2	4,089	3,671	90%	
			3	25,146	22,188	88%	
AAYL50	14 Light	6.15	1	627	552	88%	87.7%
			2	3,982	3,491	88%	
			3	25,291	22,180	88%	
AAYL51	91 Heavy	6.35	1	684	521	76%	75.2%
			2	4,141	3,131	76%	
			3	25,075	18,820	75%	
AAYL52	95 Light	6.82	1	551	548	99%	99.7%
			2	3,755	3,743	100%	
			3	25,594	25,526	100%	

#### AlphaSeq data collection

##### Yeast media

Yeast peptone dextrose (YPAD), yeast peptone galactose (YPAG), and synthetic drop out (SDO) media supplemented with 80 mg/mL adenine were made according to standard protocols. Suppliers used for our yeast media are as follows: Bacto Yeast Extract (Life Technologies), Bacto Tryptone (Fisher BioReagents), Dextrose (Fisher Chemical), Galactose (Sigma-Aldrich), Adenine (ACROS Organics), Yeast Nitrogen Base w/o Amino Acids (Thermo Scientific), SC-His-Leu-Lys-Trp-Ura Powder (Sunrise Science Products), Yeast Synthetic Drop-out Medium Supplements (Sigma-Aldrich), L-Histidine (Fisher BioReagents), L-Tryptophan (Fisher BioReagents), L-Leucine (Fisher BioReagents), Uracil (ACROS Organics), and Bacto Agar (Fisher BioReagents).

##### Isogenic yeast transformation

AlphaSeq compatible plasmids encoding yeast surface display cassettes were constructed by Twist Bioscience and resuspended at 100 ng/µL. 100 ng of plasmid was digested with PmeI enzyme for 1 hr at 37 °C to linearize, leaving chromosomal homology for integration into the ARS314 locus at both the 5′ and 3′ ends as previously described^18^. Yeast transformations were performed with Frozen-EZ Yeast Transformation Kit II (Zymo Research) according to manufactures instructions. Yeast were plated on SDO-Trp plates and grown at 30 °C for 2–3 days. Successful transformants were struck out onto YPAD plates and grown overnight at 30 °C.

##### Protein expression validation – Flow cytometry

Yeast were inoculated in YPAD and grown overnight at 30 °C. Yeast were labelled with FITC-anti-C-myc antibody (Immunology Consultants Laboratory, Inc.) in PBS (Gibco) + 0.2% BSA (Thermo Fisher Scientific) for 30 minutes at RT. Yeast were pelleted and resuspended in PBS + 0.2% BSA and read on a LSRII cytometer.

##### DNA library construction

A 300 bp oligonucleotide pool synthesized by Twist Bioscience was resuspended at 20 ng/µL in molecular grade water. Libraries were PCR amplified from the oligonucleotide pool using KAPA DNA polymerase (Roche). The oligonucleotide amplification fragment was inserted into the seed scFv backbone using Gibson isothermal assembly (NEB), as well as a second DNA fragment containing a randomized DNA barcode. The assembled barcoded antibody DNA library was PCR amplified. Fragments were run on a 0.8% agarose gel and extracted using Monarch Gel Purification kit (NEB).

##### Yeast library transformation

MATa AlphaSeq yeast were grown for 6 hours in YPAG media to induce SceI expression, as described previously^18^. All spin steps were performed at 3000 RPM for 5 minutes. Yeast were spun down and washed once in 50 mL 1 M Sorbitol (Teknova) + 1 mM CaCl_2_ solution. Washed yeast were resuspended in a solution of 0.1 M LiOAc/1 mM DTT and incubated shaking at 30 °C for 30 minutes. After 30 minutes, yeast were spun down and washed once in 50 mL 1 M Sorbitol + 1 mM CaCl_2_ solution. Yeast were resuspended to a final volume of 400 µL in 1 M Sorbitol + 1 mM CaCl_2_ solution and incubated with DNA for at least 5 minutes on ice. Yeast were electroporated at 2.5 kV and 25 uF (BioRad). Immediately following electroporation, yeast were resuspended in 5 mL of 1:1 solution of 1 M Sorbitol:YPAD and incubated shaking at 30 °C for 30 minutes. Recovered yeast cells were spun down and resuspended in 50 mL of SDO-Trp media and transferred to a 250 mL baffled flask. 20 µL of resuspended cells were plated on SDO-Trp to determine transformation efficiency. Both the flask and plate were incubated at 30 °C for 2–3 days. After 2–3 days, transformation efficiency was determined by counting colonies on the SDO-Trp plate.

##### Nanopore barcode mapping

Genomic DNA from yeast libraries was extracted using Yeast DNA Extraction Kit (Thermo Fisher Scientific) following the manufacturer’s instructions. A single round of qPCR was performed to amplify a fragment pool from the genomic DNA containing the gene through the associated DNA barcode. qPCR was terminated before saturation to minimize PCR bias, generally between 15–20 cycles. The final amplified fragment was concentrated with KAPA beads, quantified with a Quantus (Promega), prepped with a SQK-LSK-110 ligation kit (Oxford Nanopore) and sequenced with a Minion R10 flow cell (Oxford Nanopore) following the manufacturer’s instructions. Each sequencing read was aligned to the set of expected antibody sequences from the *in silico* antibody library using BLASTN^22^ to determine the mapping between DNA barcodes and antibody sequence; only DNA barcodes with at least 2 reads observed were considered, and each DNA barcode was matched to the most common BLASTN antibody match among its constituent reads.

##### Library-on-library AlphaSeq assays

Two mL of saturated MATa and MATalpha library were combined in 800 mL of YPAD media and incubated at 30 °C in a shaking incubator. Three technical replicates were performed for each assay (Table 3). After 16 hr, 100 mL of yeast culture was washed once in 50 mL of sterile water and transferred to 600 mL of SDO-lys-leu with 100 nM ß-estradiol (Sigma) for 24 hr at 30 °C in a shaking incubator. After 24 hr, 100 mL of yeast was transferred to fresh SDO-lys-leu with 100 nM ß-estradiol for an additional 24 hr at 30 °C in a shaking incubator. In addition to the antibody libraries described above, control yeast strains comprising a small network of BCL2-family proteins as previously described^18^ were included in each experiment to act as a set of standards for which BLI-derived interaction affinities were known *a priori*.

**Table 3:** Composition of AlphaSeq assays.

Assay	MATa library	MATalpha Library	No. Replicates
1	AAYL49, AAYL50	Target, Neg Ctrl 1, Neg Ctrl 2, Neg Ctrl 3	3
2	AAYL51, AAYL52	Target, Neg Ctrl 1, Neg Ctrl 2, Neg Ctrl 3	3

##### Library preparation for next-generation sequencing

Genomic DNA was extracted using Yeast DNA Extraction Kit (Thermo Fisher Scientific) following manufacturer’s instructions. qPCR was performed to amplify a fragment pool from the genomic DNA and to add standard Illumina sequencing adaptors and assay specific index barcodes. qPCR was terminated before saturation to minimize PCR bias, generally between 23–27 cycles. The final amplified fragment was concentrated with KAPA beads, quantified with a Quantus (Promega), and sequenced with a NextSeq 500 sequencer (Illumina).

##### AlphaSeq bioinformatics

Sequencing data were analyzed to identify the MATa and MATalpha barcode pairs present among diploid yeast. The observed number of sequencing reads for each MATa/MATalpha combination were normalized according to frequency among haploid yeast to account for uneven distribution of the input populations. Each aα pair was then assigned a score representing the ratio of observed sequencing reads to expected sequencing reads assuming random mating. A linear regression was performed comparing these normalized sequencing scores to known affinities for the control yeast strains and this regression was utilized to assign estimated affinities to all other aα pairs for each mating replicate.

## Data Records

### Data structure and repository

A single dataset was generated during this study. This data set contains the output of two AlphaSeq assay performed as part of a single study and is deposited at Zenodo^23^ (10.5281/zenodo.5095284). The dataset contains the variables listed in Table 4.

**Table 4:** Variables and associated descriptions.

Variable Name	Description
POI (Protein of Interest)	Alphanumeric label corresponding to amino acid sequence
Sequence	Single letter amino acid representation of scFv measured.
Target	Protein target represented by a text label for which the measured antibody interacted with. Options are defined target or negative controls 1–3.
Assay	Unique assay identifier, either 1 or 2
Replicate	Unique replicate identifier, either 1, 2 or 3
Pred_affinity	Value representing the score from the AlphaSeq assay, as described in the methods section. These values estimate the protein-protein dissociation constant in nanomolar, on a log scale, and are the result of empirical measurement. Lower values indicate stronger binding.
HC, LC	Single letter amino acid sequence of the heavy chain (HC) or light chain (LC)
CDR[H/L][1/2/3]	Single letter amino acid sequence of a CDR region where H indicates heavy chain, L indicates light chain and the numerical value represents either CDR 1, CDR 2 or CDR 3.

### Data sets and file types

The data are stored in a single.csv file. All data can be downloaded from Zenodo^23^.

## Technical Validation

### Library coverage

To ensure sufficient proportions of designed sequences were assembled into each library and to confirm no incorporation bias based on number of mutations, we evaluated the percentage incorporation per *k* mutations per library (Table 2). The intra-library variation is small, never exceeding 2%, indicating no bias due to number of mutations. Because each library is constructed separately, it is expected that the inter-library differences in incorporation will be greater than that of the intra-library range. This is the case with incorporation percentages ranging from 75.2% to 99.7%. Given these observations, we conclude the libraries are constructed sufficiently.

### Reproducibility

**Fig. 2:**
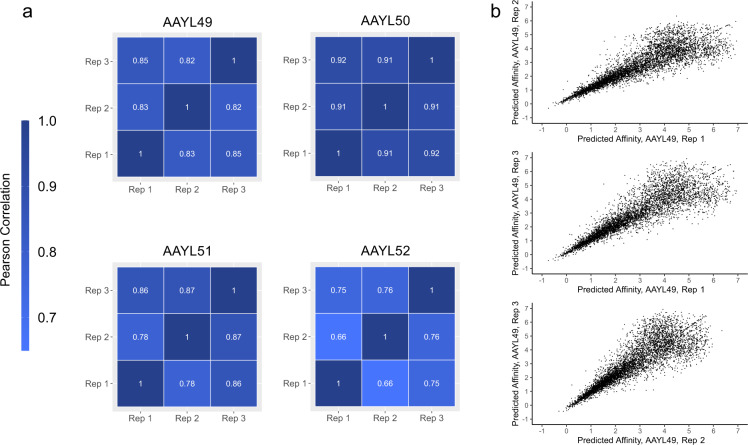
Reproducibility of AlphaSeq measurements. (**a**) Pearson correlation among technical replicates for each of the four libraries. Darker blue represents greater correlation. (**b**) Pairwise comparison for each pair of replicates from library AAYL49. Sequences without replicate analyses are not plotted.

To assess variation attributable to the AlphaSeq process, each yeast mating experiment was performed in triplicate, with separate determination of K_d_ values for each technical replicate. Figure 2a includes matrices with pairwise Pearson Correlation values for each pair of replicates within a given library. The Pearson Correlation ranges from 0.66 (AAYL52 Rep 1 vs Rep 2) to 0.93 (AAYL50 Rep 1 vs Rep 3). Figure 2b presents a visualization of the pairwise comparison of each replicate within AAYL49. The observed phenomena of better correlation at lower predicted affinity values holds true across each library. Affinity measurements, especially sub-micromolar affinities, are highly reproducible between AlphaSeq replicates.

### Analysis of standards

**Fig. 3:**
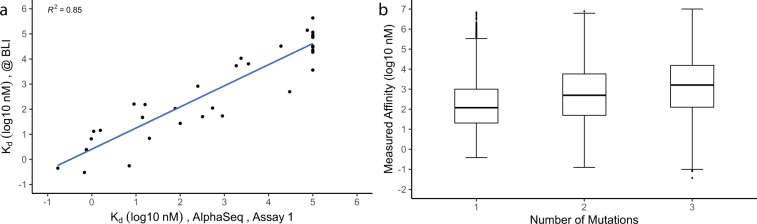
Analysis of standards and identification of expected data patterns. (**a**) Correspondence between known K_d_ values and AlphaSeq-predicted affinity values for a known PPI network. (**b**) Box-and-whisker plot showing the distribution of AlphaSeq-predicted affinity values for each variant against the target, binned by number of mutations within the antibody sequence.

Control yeast strains comprising a small network of BCL2-family proteins, as previously described^24,25^ were included in each experiment to act as a set of standards for which bio-layer interferometry-derived interaction affinities were known *a priori*. Figure 3a shows the correspondence between known K_d_ values and AlphaSeq-predicted affinity values for these PPIs, with a computed linearity of R^2^ = 0.85.

### Analysis of binding affinity for 1/2/3-site variants

To further validate the assay results by identifying expected patterns, AlphaSeq-derived binding affinities were compared for all antibody sequences, binned by the number of mutations separating each antibody from its seed sequence (1, 2 or 3). Results are shown in Fig. 3b; as expected, median affinity decreases with each additional mutation (2.08 log_10_ nM, 2.70 log_10_ nM, 3.21 log_10_ nM respectively for 1, 2, 3 mutations) while variance increases with each additional mutation (interquartile range 1.69 log_10_ nM, 2.06 log_10_ nM, 2.09 log_10_ nM). In other words, each added mutation increases the probability of breaking the antibody but there is also more room for improvement over the wild type.

## Usage Notes

### Binding to negative controls

The inclusion of MATα yeast expressing no POI serves as an opportunity to identify MATa yeast with non-specific binding. These negative control yeast strains: AlphaNeg1, AlphaNeg2, and AlphaNeg3 are expressing AGA2 with an N-terminal HA epitope tag and C-terminal Myc tag without a POI. As such, many of the entries within the dataset represent interactions between MATa yeast with a MATα yeast expressing a negative target. The values associated with these measurements range from 1.03 log_10_ nM to 7.14 log_10_ nM for assay 1 and 1.35 log_10_ nM to 7.32 log_10_ nM for assay 2. As these values are higher than the distribution of values for on-target binding, they serve as an additional confirmation that the pred_affinity measurements are resultant of on-target binding. The binding affinities measured against negative controls represent some combination of nonspecific yeast mating and molecular artifacts introduced to the barcodes during PCR and sequencing and can act as an empirical readout of the limit of blank for this dataset. Note that given the increase in technical variation observed with increasing pred_affinity values, it is not recommended to background subtract these values from the on-target pred_affinity measurements.

### Normalization among replicates and assays

Each assay and replicate contains each of the three seed sequences and can be used to normalize the data among the assays or replicates. These sequences can also be included in future assays to allow for integration of additional data. Additionally, the regression used to transform sequencing abundances to predicted affinity values is performed once for each replicate and then applied to the entire replicate; relative ranking of interactions within a replicate are insensitive to any technical variation in that calculation, but such error will propagate to all quantitative predicted affinity measurements in that replicate.

### Sequences without a pred_aff value

Data entries in which a sequence and target pair is specified but does not have a pred_aff value indicate a poor binding interaction. These antibody sequences are observed in DNA sequencing of the MATa haploid yeast population, but not among diploid yeast, affirming the sequence is present in the MATa library but no mating was observed. There are multiple options for how to treat these entries in downstream applications, including removing them from the dataset. While not conclusive, absence of diploids is strong evidence of poor binding affinity; imputing affinity values to indicate as such may be advantageous. Values could be imputed, for example, as the maximum pred_aff value or as the median pred_aff value of sequences not having measurements in all replicates.

## Data Availability

Code associated with the randomization of antibody designs is available on GitHub (https://github.com/mit-ll/Insilico_Ab_Variant_Generator). Code used for sequence analysis is functionally similar to code that has previously been released^18^. This code can be accessed on GitHub (https://github.com/dyounger/yeast_synthetic_agglutination).
